# The Grand Challenges of Medical Technology

**DOI:** 10.3389/fmedt.2020.00001

**Published:** 2020-07-15

**Authors:** Alicia J. El Haj

**Affiliations:** Healthcare Technology Institute, Institute of Translational Medicine, University of Birmingham, Birmingham, United Kingdom

**Keywords:** medical technology, healthcare, biomedical engineeirng, healthcare innovation, enabling technologies, regenerative technology

## The Vision

New developments in medical technology span the breadth of the healthcare environment providing new solutions for therapies, diagnostics, and imaging. Healthcare is a billion dollar market which drives the development and progression of healthcare technologies to the clinic. The innovative approaches which involve novel tools and technologies form the basis for this translation. Exciting areas highlighted by this new Journal, such as Cardiovascular Medtech, Nano-Based Drug Delivery, Medtech Data Analytics, Pharmaceutical Innovations, Regulatory Affairs and Regenerative Technologies represent key examples where step changes in healthcare solutions are being addressed. In each one of these sectors and across sectors, there are major challenges which we can identify.

Probably the biggest underlying global challenge which requires a new “mind set” is maintaining a high quality of life for our aging populations. Across the globe, people are living longer and requiring new solutions to address life as an aged citizen. Regenerative technologies and robotics are highlighting a new era of how we maintain a healthy life through a millennial lifespan. Soft robotics and tissue engineering provide potential reparative solutions. New innovative technologies can help to maintain healthy lives in our aging populations by addressing major chronic and acute clinical conditions. Scientific disciplines in biology and molecular approaches have revealed the potential for new drugs and treatments, however, there are still significant gaps in the enabling technologies and supporting medical technology devices which need addressing. Recent viral pandemics such as COVID 19 have demonstrated clearly the need for new medical technologies to fight disease such as portable ventilators and respiratory assistive devices useable throughout the world in developed and underdeveloped countries. These future technologies require the skillsets of a diverse academic base crossing many scientific and engineering communities. In addition, to bring new approaches to the clinic, many facets of the field must be determined and defined to the standards and rigor of the scientific, regulatory and clinical communities. With the increase in innovative technologies and regenerative therapies aiming for the clinic, there are key challenges in delivery systems, metrology, quantitative and computational modeling, data throughput, multimodal approaches for the characterization of disease and treatments, physiological and biochemical monitoring of clinical treatments and tools, technology integration and automation.

## What Are Examples of the Major Challenges We Face in the Med Tech Sector?

### Scale

Over the past 10 years, the scale of healthcare therapies is reducing with “nanotechnologies” providing exciting opportunities. Examples of nanotechnologies include drug delivery systems with smaller and more targeted approaches, nanomagnetic solutions with nanoparticles for imaging and treatments such as hyperthermia in cancer. The ability to modify materials rapidly at small length scales using techniques such as laser direct printing and other 3D printing modalities provides opportunities for unique capabilities in the fabrication of medical devices (1). Techniques such as laser and 3D printing provide opportunities for processing a broad spectrum of advanced medical devices, such as drug delivery devices, stents, patient-specific prostheses, biosensors, and regenerative technologies (2).

### Biomedical Complexity

Coping with the complexity of tissues and organs alongside the issues of multi morbidity in patients requires new approaches in targeting and specificity of drugs and other medical devices. Design for additive manufacturing (DfAM) aims to utilize the complexity of human systems for the development of medical devices. DFAM provides enhanced performance in our ability to generate biomaterials with complex geometrical designs at the micro-scale (3). New nano—drug release strategies allow localized delivery to provide specific solutions to regions and organs in the body (4).

### Personalized and Bespoke Medicine

Stratification of patient populations is presenting a new era of personalized medicine. How we stratify patients requires new tools and diagnostic capabilities (4). How we tailor treatments to improve efficacy requires autologous and synthetic treatments. Repurposing existing drugs and new multi model drug designs require innovations in computational and *in silico* tools (5). The pharmaceutical industry is having to rethink how next generation drugs are developed and delivered enabling more personalized approaches within populations.

### Early Diagnostics

Early screening for disease prevention rather than end stage treatment of degeneration has been proposed as a key solution for us to tackle long term conditions (6). New advances in the treatment of chronic disease require early identification often prior to detection of major symptoms. A disease which illustrates this challenge is Osteoarthritis where treatment is often given when the joint has completely failed and requires replacement. Early interventions which can arrest the degeneration of the joint would eradicate the need for these major surgeries. New diagnostic and imaging techniques are being developed which support early screening programmes.

### Precision and Robotic Surgery

Surgical procedures in cardiovascular, opthamology and other major organs are evolving and improving with clinically validated protocols. Precision surgical tools combined with robotics and virtual surgeries provide the supporting technology advances in this area. New technologies have reached the clinic in minimally and non-minimally invasive, transluminal endoscopic, and single site surgeries (7).

### Rehabilitation and Assisted Devices

Innovations in assisted devices are building mobility in our physically impaired populations. Advances in materials, electronics and designs are revolutionizing our ability to support and mobilize this community. The role of tissue mechanics and mechanotransduction has been identified in rehabilitation and provides a future area for new forms of rehabilitation regenerative therapies (8).

### Stem Cells for Regeneration and Therapy

Stem cell therapies and regenerative medicine require significant support in the form of enabling technologies to reach the clinic. To deliver regenerative medicine therapies involves scalable production and application of standardized clinical grade biotherapies. The delivery is underpinned by effective supply chain capabilities combined with manufacturing and sourcing (9). These enabling technologies such as bioreactors for growing cells and biomaterial systems are identified within a new field termed regenerative medical technologies. In addition, novel diagnostics which monitor cell performance and efficacy are required to support regulatory approvals (10).

### The Regulatory Environment and Standardization

New challenges include how to assess advances in therapies using health technology assessments which are tailored for new fields such as gene therapy or cell therapies. The expansion of medical technology in our hospitals has not kept pace with patient safety. Assessments and safety requirements play an important role in protecting against risk of failure. Evidence suggests that pioneer entrants in medical technology may take significantly longer than follow on products which can be related to novel assessments and lack of regulatory guidelines in some cases (11).

### Big Data

Finally, large databases of patient data are being captured in hospitals which if accessed provide wealth of information about disease treatment and prevention. Handling data sets and analysis of data has become a major growth area of interest globally (12). Mobile medical technology is expanding with multiple diagnostic and monitoring platforms using mobile app systems which can require new ways of approaching data analytics.

## Conclusions

To tackle these challenges, the Med Tech field needs to work as a community celebrating their new high quality approaches and integrating them across organ sectors. This new journal, Frontiers in Medical Technology, will provide a forum for these disparate communities to come together and publish their articles in an open access format for dissemination. Reaching out to the clinician, clinical scientist, academic, engineering and commercial worlds, we aim to provide an on-line forum for high quality pier reviewed publications which spearheads new innovations in a global field and demonstrates their relevance in a clinical setting.

## Author Contributions

The author confirms being the sole contributor of this work and has approved it for publication.

## Conflict of Interest

The author declares that the research was conducted in the absence of any commercial or financial relationships that could be construed as a potential conflict of interest.
